# Perturbation of *Brachypodium distachyon CELLULOSE SYNTHASE A4* or *7* results in abnormal cell walls

**DOI:** 10.1186/1471-2229-13-131

**Published:** 2013-09-11

**Authors:** Pubudu P Handakumbura, Dominick A Matos, Karen S Osmont, Michael J Harrington, Kyuyoung Heo, Kabindra Kafle, Seong H Kim, Tobias I Baskin, Samuel P Hazen

**Affiliations:** 1 Biology Department, University of Massachusetts, Amherst, MA, USA; 2 Plant Biology Graduate Program, University of Massachusetts, Amherst, MA, USA; 3 Molecular and Cellular Biology Graduate Program, University of Massachusetts, Amherst, MA, USA; 4 Department of Polymer Science and Engineering, University of Massachusetts, Amherst, MA, USA; 5 Department of Chemical Engineering, Pennsylvania State University, University Park, PA, USA

**Keywords:** Artificial microRNA, *Brachypodium distachyon*, Crystallinity, Secondary cell wall, Sum-frequency-generation vibration spectroscopy, Xylem

## Abstract

**Background:**

Cellulose is an integral component of the plant cell wall and accounts for approximately forty percent of total plant biomass but understanding its mechanism of synthesis remains elusive. CELLULOSE SYNTHASE A (CESA) proteins function as catalytic subunits of a rosette-shaped complex that synthesizes cellulose at the plasma membrane. *Arabidopsis thaliana* and rice (*Oryza sativa*) secondary wall *CESA* loss-of-function mutants have weak stems and irregular or thin cell walls.

**Results:**

Here, we identify candidates for secondary wall *CESA*s in *Brachypodium distachyon* as having similar amino acid sequence and expression to those characterized in *A. thaliana*, namely *CESA4*/*7*/*8*. To functionally characterize *BdCESA4* and *BdCESA7*, we generated loss-of-function mutants using artificial microRNA constructs, specifically targeting each gene driven by a maize (*Zea mays*) ubiquitin promoter. Presence of the transgenes reduced *BdCESA4* and *BdCESA7* transcript abundance, as well as stem area, cell wall thickness of xylem and fibers, and the amount of crystalline cellulose in the cell wall.

**Conclusion:**

These results suggest *BdCESA4* and *BdCESA7* play a key role in *B. distachyon* secondary cell wall biosynthesis.

## Background

With continued consumption of fossil fuels, humankind faces a growing challenge of finding renewable sources of energy. Although plant derived biomass contains appreciable energy, to serve as a fuel for transportation it must be chemically or biologically liquefied, a conversion that is the subject of strenuous efforts to make economical. Success will presumably require not only advances in chemistry such as better catalysts, but also improved plants to serve as feedstock. As an ideal input, grasses are receiving considerable attention because certain species grow to great density, are perennial, and can require little if any fertilizer or irrigation [1]. But their very size, longevity, and genome complexity makes these species difficult subjects to study and breed.

For studying grasses, whether as sources for biofuel or for any other grass-specific question, an emerging model is *Brachypodium distachyon.* This species is closely related to cereals and temperate grasses, and is about the same size as *Arabidopsis thaliana* and has about the same generation time. Studies on *B. distachyon* benefit from rapidly developing community resources, such as a completely sequenced genome, mutant collections (both chemically derived and sequence-indexed DNA insertions), and efficient protocols for transformation and crossing [2-5]. Of particular note for biofuels research, *B. distachyon* has cell walls that are similar compositionally to that of other grasses, like wheat (*Tritium aestavum*), barley (*Hordeum vulgare*), and *Miscanthus*[6-8]. Insofar as the cell wall constitutes almost the entirety of the input for converting biomass to biofuel, this similarity, along with the genetic attributes of this small grass, emphasize its suitability as a model for grass-related, biomass crop research.

Within the cell wall, as a target for optimization, cellulose is pre-eminent. Cellulose is the most abundant of any single wall component and is made exclusively of glucose, a tractable and energy rich molecule. Cellulose comprises long polymers of (1–4) β-linked glucose that are synthesized at the plasma membrane and associate laterally into a microfibril. Because of the configuration of the glucose residues, hydrogen bonds form at great density both within and between chains, a density that gives cellulose an elastic modulus rivaling that of steel but makes the structure impervious to degradation whether chemical or enzymatic.

Within the plasma membrane, the structure synthesizing cellulose is called a “terminal complex” [9-11]. In land plants and related green algae, the terminal complex as seen in the electron microscope comprises six subunits with hexagonal symmetry and is called a “rosette” [12]. The primary constituents of the rosette are CELLULOSE SYNTHASE A (CESA) proteins. These proteins belong to processive glycosyltransferase family 2 and are thought to be the catalytic subunits for polymerizing the glucose chain. In angiosperms, CESAs usually comprise a small gene family with around ten members [13,14].

Identification of CESA proteins and characterization of their function has greatly benefited from the facile genetics of *A. thaliana*. From this work, it emerged that certain CESA proteins synthesize the primary cell wall whereas others synthesize the secondary cell wall [15,16]. Furthermore, it appeared that a given cell must express three distinct CESA proteins to produce cellulose at optimal levels. For the secondary cell wall, a screen based on collapsed xylem cells led to the identification of several *irregular xylem* (*irx*) lines, three of which, *irx5*, *irx3*, and *irx1*, harbor lesions in AtCESA4, 7, and 8, respectively [17-19]. Supporting the hypothesis of non-redundancy, these genes are expressed at similar levels in similar cell types, and the null mutants have indistinguishable phenotypes, including weak stems, collapsed xylem, and thin secondary cell walls that are deficient in cellulose.

Identification of a trio of CESA genes primarily responsible for synthesizing cellulose in secondary cell walls has been supported by work in other systems, including grasses. First, AtCESA4, 7, and 8 are represented in some angiosperm species by a single sequence each, and orthologs are more closely related than homologs (i.e., CESA4s of various species resemble each other more closely than do CESA4, 7, and 8 of a single species) [13,20,21]. Second, in grasses, a mutant of barley, *fragile stem 2*, with brittle stems and low cellulose content in the mature plant was attributed to a lesion in the barley ortholog of AtCESA4 [22]. In addition, “*brittle culm*” mutants in rice (*Oryza sativa*) have been mapped to the three orthologs of AtCEA4, 7 and 8 and again these mutants have similar phenotypes, including modest dwarfism, thinner and weaker culms, and reduced cellulose content [23]. However, it is not understood why three distinct proteins are needed nor is it known which of the loss-of-function phenotypes result directly from the lost protein and which result as a consequence of cumulative effects.

Here we describe the *CESA* gene family in *B. distachyon* with the aid of gene expression profiling and phylogeny. Furthermore a detailed analysis of the candidate secondary CESAs were performed by functionally characterizing mutants generated with specific artificial microRNA constructs.

## Results

### *Brachypodium distachyon* CESA gene family

**Figure 1:**
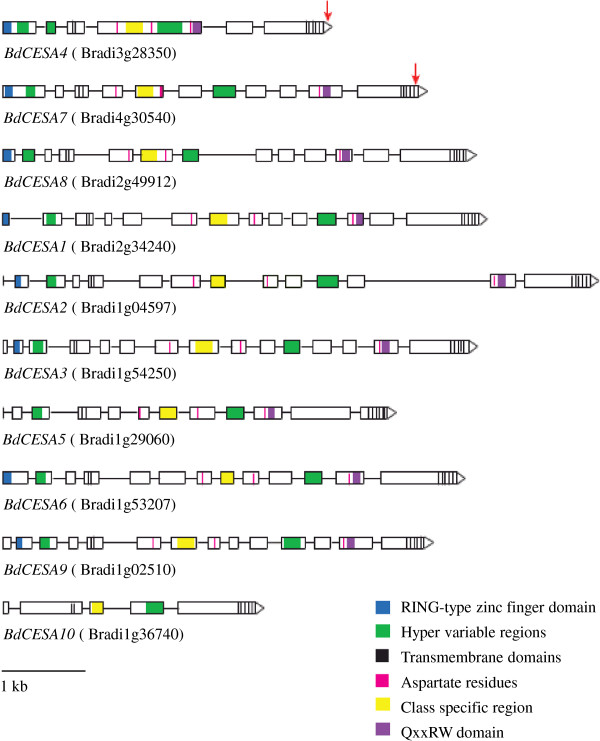
**Models of *Brachypodium distachyon CESA *genes.** Exons are indicated by boxes and introns by lines. Genes are drawn to scale; the bar in the lower left indicates 1 kb. Red arrows indicate regions used for artificial microRNA targeting.

In *B. distachyon,* as in *A. thaliana* and rice, the CESA family comprises ten genes (Figure 1). Their genomic sequences range from 3045 to 7601 bp, with 5 to 14 exons that form coding regions ranging from 2331 to 3279 bp (776 to 1092 amino acids). Amino acid sequence comparisons revealed extensive similarity among the BdCESA proteins, with conserved structural features that are characteristic of CESA protein families [13,14]. All ten BdCESA proteins contain eight transmembrane domains and two hyper-variable regions. With the exception of BdCESA10, they have the signature motif of glucosyl transferases, D,D,D,QxxRW, which is essential for binding UDP-Glucose. With the exception of BdCESA5 and 10, the proteins contain the RING-type zinc finger domain with eight cystine residues spaced as characteristic for CESA proteins. Even though BdCESA10 is categorized as a CESA, it is short, and lacks the RING-type zinc finger motif, a portion of the first hyper-variable region, and two of the conserved aspartate residues of the QxxRW motif. This is also true of OsCESA11 and sorghum (*Sorghum bicolor*) Sb10g023430*,* both of which are nonetheless considered part of the CESA family [24].

**Figure 2:**
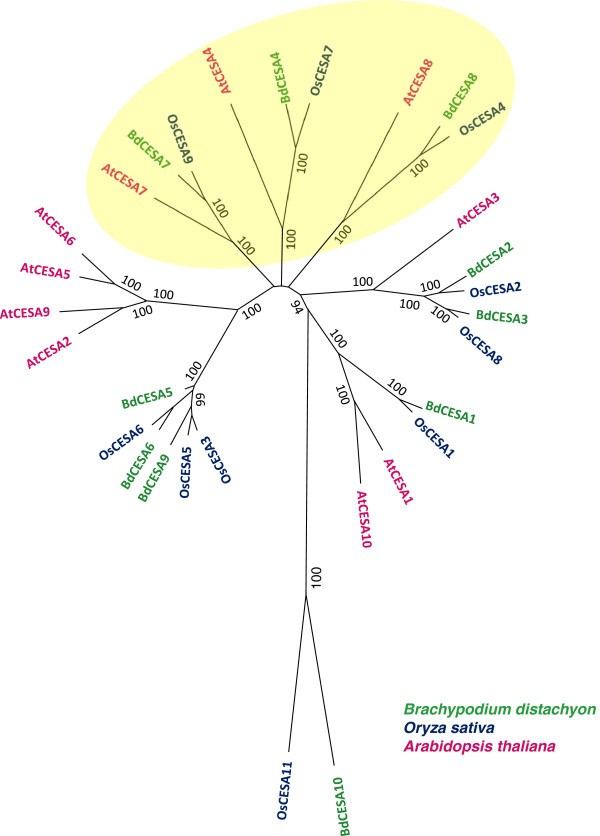
**Phylogenetic analysis of CESA amino acid sequences.** Numerical values on branches refer to neighbor-joining bootstrap support. Yellow oval denotes proteins associated with secondary cell walls.

Based on the amino acid sequence, the ten BdCESA proteins fall into reasonably well-established phylogenetic groups (Figure 2). This is clearest for proteins associated with the secondary cell wall. *Brachypodium distachyon* has three sequences that are highly similar to those characterized in other species as secondary cell wall CESAs. We numbered these genes *BdCESA4*, *BdCESA7*, and *BdCESA8* based on their apparent orthologs in *A. thaliana*. Note that the published numbering differs for rice. While the three secondary CESA clades have a single member in each species, there is no complete one-to-one relationship for the CESAs associated with the primary cell wall. For example, *B. distachyon* has a single sequence in the CESA1 clade whereas *A. thaliana* has two. In contrast, *B. distachyon* has two sequences in the CESA3 clade whereas *A. thaliana* has only one. Interestingly, both *B. distachyon* and *A. thaliana* have several members of the CESA6 clade but these duplications appear to have formed in each lineage after the divergence of the two species. Based on sequence and conserved domains BdCESA10 seems to be the least similar to other CESA proteins. However a comprehensive phylogenetic analysis of the Cellulose synthase A proteins and Cellulose Synthase like proteins of *A. thaliana*, rice and *B. distachyon* reveled the distinct similarity of BdCESA10 to other CESA proteins (Additional file 1: Figure S1). Where possible, we numbered the *B. distachyon* gene after its closest relative in *A. thaliana*.

In view of our interest in the secondary cell wall, we examined the *B. distachyon* secondary cell wall sequences at greater depth (Additional file 2: Figure S2). In the cystine rich RING-type zinc finger domain, the sequences differ in the spacing between the second and third cystine. BdCESA4 has the canonical 15 amino acids whereas BdCESA7 has a single amino acid insertion and BdCESA8 has an eight amino acid deletion. Such spacing variations have apparently not been previously reported for *A. thaliana* secondary CESAs. However, the rice secondary CESA homologous to BdCESA8 exhibits the same eight amino acid deletion [23]. The RING-type zinc finger domain is the distinguishing feature of CESA proteins seen in the first portion of the N-terminus and is thought to be involved in CESA protein dimerization [10,11].

### *Brachypodium distachyon* secondary cell wall CESA gene expression

**Figure 3:**
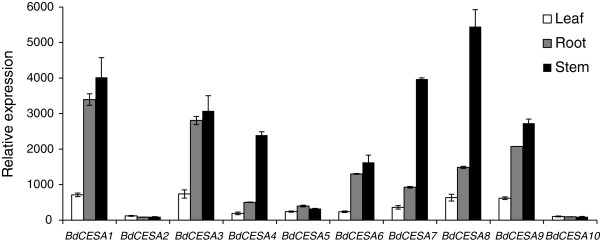
**Relative abundance of *CESA *transcripts in different organs measured with a microarray.** Bars plot mean ± standard deviation of three biological replicates.

To analyze *CESA* gene expression, we profiled transcripts with a whole genome tiling array focusing on organs expected to be enriched in secondary cell wall synthesis. To obtain RNA, leaves and stems were harvested when the inflorescence emerged from the flag leaf, whereas roots were harvested from seven-day-old seedlings. Additionally, to minimize changes in transcript abundance due to diurnal and circadian rhythms, RNA was pooled from material harvested at six different circadian time points over a 24-hour period. In all three organs, *BdCESA2* and *10* were expressed at essentially background levels and *BdCESA5* was slightly greater (Figure 3). The four genes associated with the primary wall, *BdCESA1, 3*, *6*, and *9*, were expressed at high levels in both root and stem. On the other hand, those associated with the secondary wall were expressed at much lower levels in roots than stems, having a ratio of expression roughly consistent with secondary cell wall content across the three organ types (Additional file 3: Table S1).

### Localization of putative secondary cell wall CESA transcripts

**Figure 4:**
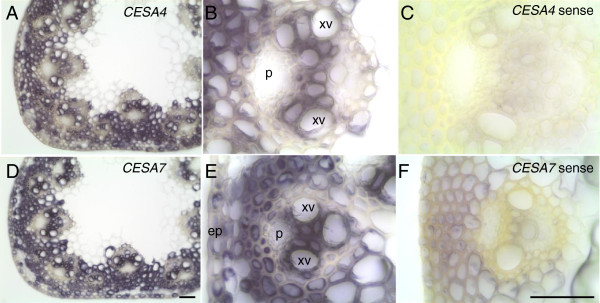
***BdCESA4 *and *BdCESA7 *are expressed in cells undergoing secondary wall deposition in the stem.** Expression of *CESA4***(A, B)** and *CESA7***(D, E)** analyzed by *in situ* hybridization at three weeks of development when the inflorescence was just emerging from the flag leaf. Cross sections through the first internode were labeled with anti-sense probes, imaged at 10x **(A, D)** and 40x **(B, E)**; and sense probes, imaged at 40x **(C, F)**. xv, xylem vessel; p, phloem; ep, epidermis; Scale bar = 50 μm.

To localize transcripts, we performed RNA *in situ* hybridization on stems (Figure 4). Stems were fixed, sectioned on a Vibratome, and hybridized with labeled sense and anti-sense probes. Upon color development, positive hybridization was detected mainly in the sections probed with the anti-sense probes. Consistent with a role in secondary cell wall synthesis, hybridization for each probe was strong in the vascular bundles and the surrounding mechanical cells including the sclerenchyma fibers and the epidermis. Hybridization was essentially undetectable in pith parenchyma, which undergoes limited cell wall thickening. These results strengthen the assignment of *BdCESA4* and *BdCESA7* as secondary cell wall related CESAs.

### Artificial microRNAs targeting BdCESA4 and BdCESA7

**Figure 5:**
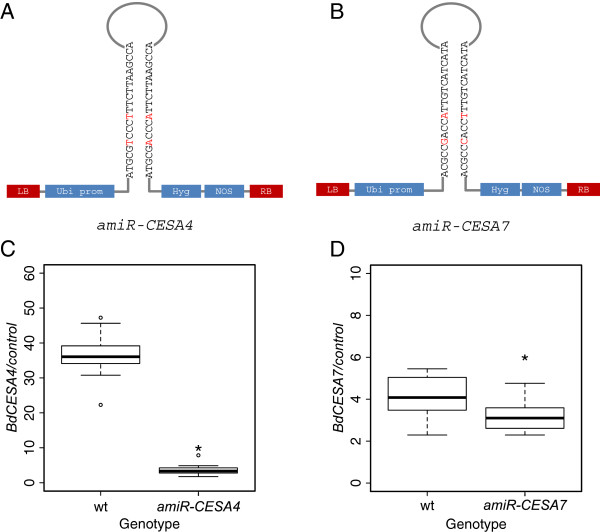
**Targeting *CESA *expression by means of artificial microRNAs. (A, B)** Schematic of the constructs used. The hairpin model illustrates the 21-mer sequence of each microRNA construct and red letters indicate the mismatch in each hairpin recognized by the DICER complex. LB, left border; Ubi prom, maize ubiquitin promoter; Hyg, hygromycin phosphotransferase gene; NOS, nopeline synthase terminator; RB, right border. **(C, D)** Relative levels of transcript measured by RT-QPCR. Reference genes are given in methods. Stem tissue was collected at the same development stage when inflorescence was just emerging from the flag leaf. Three to five individuals from three to four independent transgenic lines were analyzed for each construct. The boxes show interquartile range, the whiskers show the outer quartile edge, and the black line represents the median of each distribution. Open circles represent outliers, when present. * Denotes significance at the 5% level.

To examine the function of BdCESA4 and 7, we sought to reduce transcript levels by means of artificial microRNAs (*amiR*). The rice microRNA, *osaMIR528*, was modified to specifically target either *BdCESA4* or *BdCESA7* (Figure 5A, B). Each modified microRNA is predicted to target only a single gene. The *amiR-CESA4* construct specifically targets nucleotides 3106 to 3126, which are in the last exon immediately after the last transmembrane domain; in contrast, *amiR-CESA7* targets nucleotides 3028 to 3048, which are in the last exon in between the seventh and the eighth transmembrane domains (Figure 1). To characterize the efficacy of these constructs, we measured mRNA levels in the stem of T_3_ generation plants. For each construct, three to five individuals from families derived from three or four independent transformation events were analyzed by reverse transcriptase quantitative PCR (RT-QPCR). Stems were harvested when the inflorescence was just emerging from the flag leaf, at developmentally equivalent time points. Both of the artificial microRNA constructs significantly reduced transcript abundance of the corresponding target (Figure 5C, D). Specifically, *BdCESA4* was reduced 9.5 fold and *BdCESA7* was reduced 1.5 fold. Neither transgene detectably reduced the level of *BdCESA8* and *amiR-CESA7* caused no significant decrease in *BdCESA4*; however, *amiR-CESA4* modestly decreased the expression of *BdCESA7* (Additional file 4: Figure S3).

### BdCESA4 and BdCESA7 knock-down lines and the structure of the stem

**Figure 6:**
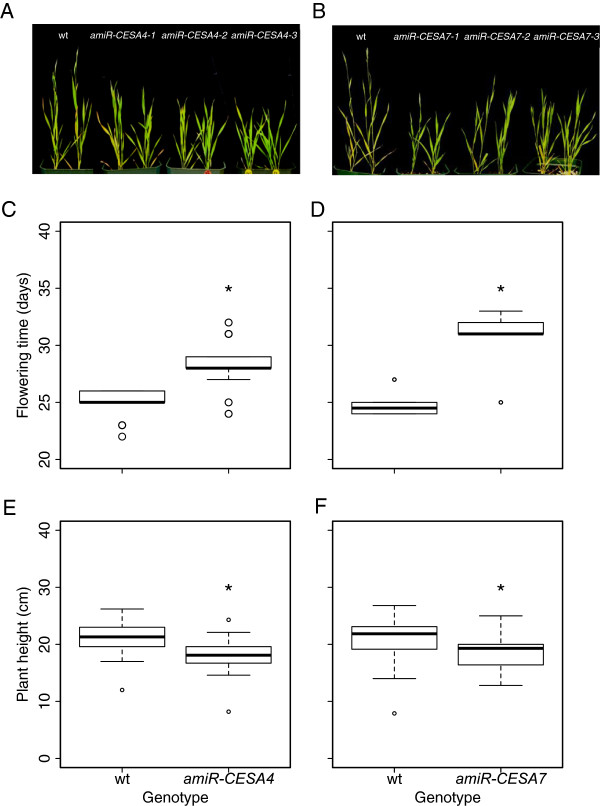
**Whole plant phenotypes. (A, B)** Plants at the time of wild-type inflorescence emergence. Representative plants of wild-type and of three independent lines used for each construct. **(C, D)** Days to inflorescence emergence. **(E, F)** Mature plant height. Twenty to thirty individuals from three independent lines were analyzed for each construct. Box plots and significance are as described for Figure 5.

**Figure 7:**
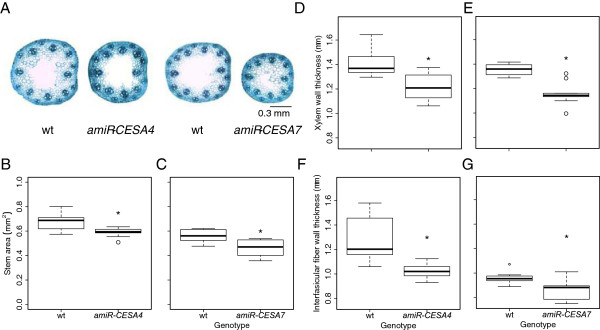
**Stem anatomy. (A)** Toluidine blue-stained transverse sections. **(B, C)** Stem area. Cell wall thickness of **(D, E)** metaxylem and **(F, G)** interfascicular fibers. First internode was collected and analyzed at inflorescence immergence from developmentally equivalent plants. Three to five individuals from three independent lines were analyzed. Box plots and significance are as described for figure 5.

The *BdCESA* knock-down lines were modestly but significantly decreased in stature and delayed in inflorescence emergence (Figure 6). To investigate anatomical changes, we examined first internode morphology of the same plants assayed for mRNA levels. Because the transgenic lines were grown at different times, each comparison included a wild-type control, and differences between these controls presumably reflect differences in growth conditions. Hand-cut transverse stem sections were stained with the polychromatic basic dye, toluidine blue, and imaged using a light microscope. Toluidine blue differentially stains cell wall polymers— polysaccharides purplish blue and lignified cell walls turquoise—allowing cell types to be distinguished. The artificial microRNA constructs had little if any effect on the overall shape and arrangement of the vascular bundles (Figure 7A). Likewise, there was no significant difference between the genotypes in number of vascular bundles in either the inner or the outer ring (data not shown). While changes in anatomy were minor or absent, stem diameter appeared to be reduced, consistent with the decreased plant height. Measurements of stem area revealed a modest reduction, but one that was significant for both *amiR-CESA4* and *amiRCESA7* (Figure 7B, C)*.*

To examine the effects of the artificial microRNA constructs on cell wall structure, we measured cell wall thickness in the toluidine blue-stained sections. For both targets, the constructs reduced the thickness of cell walls modestly, but significantly (Figure 7D-G). The reduction was similar for xylem as well as for interfascicular fibers. This observation is consistent with the phenotypes of secondary *CESA* mutants characterized in other grass species [22,23,25].

### Knock-down of BdCESA4 and BdCESA7 and crystalline cellulose content

**Figure 8:**
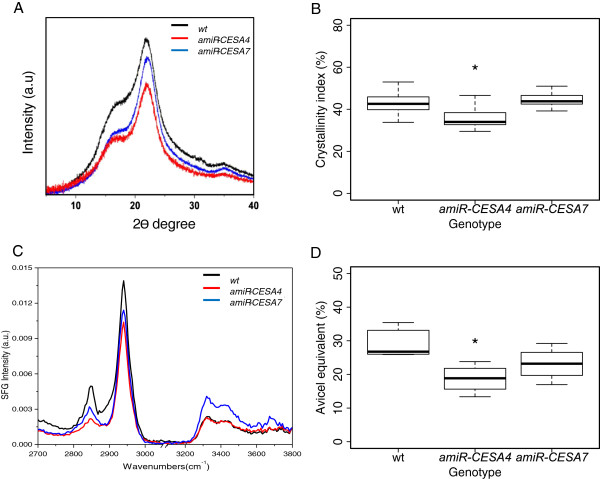
**Spectroscopic analysis of cellulose crystallinity. (A)** X-ray powder diffraction profiles. **(B)** Crystallinity index derived from the diffraction profiles using the amorphous cellulose subtraction method. **(C)** Sum-frequency-generation vibration spectra. **(D)** Cellulose crystallinity derived from the spectra based on comparison to Avicel. Eight to twelve individuals from three independent lines were analyzed for each transgene. Box plots and significance are as described for figure 5.

Reduction in stem size along with the thinner cell walls indicated the possibility of a change in cell wall structure. Completely senesced and homogenized stem tissue was analyzed. First, crystalline cellulose content was assayed by X-ray powder diffraction, using the same individuals analyzed for Figures 6 and 7, with the two wild-type samples pooled. The transgenic genotypes gave diffraction patterns with lower intensities at nearly all measured angles, although the effect in *amiR-CESA4* was stronger than in *amiR-CESA7* (Figure 8A). To evaluate cellulose crystallinity, as described in Methods, we calculated a crystallinity index by means of the so-called “amorphous subtraction method” (Figure 8B). The crystallinity index confirmed the visual impression of the diffraction patterns, namely a significant reduction in cellulose crystallinity for *amiR-CESA4*.

Second, we evaluated the amount of crystalline cellulose by sum-frequency-generation (SFG) vibration spectroscopy [26]. In this method, the sample is irradiated simultaneously by visible laser pulses at 532 nm and by infrared laser pulses with a tunable frequency. Among reflected and scattered lights, there are photons whose frequency is the sum of two input laser frequencies, which can be filtered and recorded separately. Due to the symmetry requirements, this frequency summation can be caused by crystalline cellulose but not by amorphous non-crystalline cell wall components [26]. For this analysis, lines were grown at the same time. In the spectra, there are three prominent peaks indicative of crystalline cellulose Iβ: namely 2850 cm^-1^ ascribed to symmetric CH_2_ stretching, 2944 cm^-1^ ascribed to asymmetric CH_2_ stretching, and 3320 cm^-1^ ascribed to the intra-chain hydrogen-bonded hydroxyl stretch (Figure 8C) [26,27]. Taking Avicel, a model cellulose Iβ with a high crystallinity, as a standard, the intensity at 2944 cm^-1^ can be used to estimate the crystalline cellulose amount by means of the previously determined calibration curve [27]. The comparison of the intensities recorded at 2944 cm^-1^ indicated that the Avicel-equivalent crystalline cellulose content tended to be reduced in *amiR-CESA7* and was significantly reduced in *amiR-CESA4* (Figure 8D). These results are comparable to those from X-ray diffraction.

**Figure 9:**
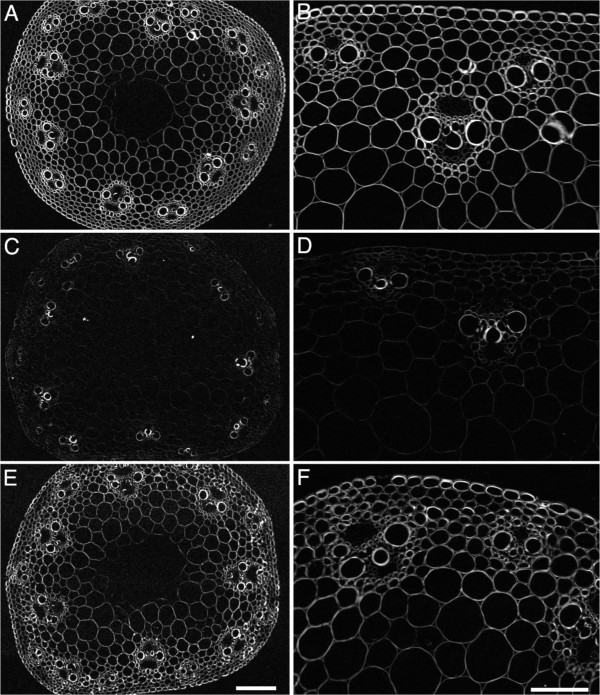
**Polarized-light micrographs of stem internode transverse cross-sections.** Representative images of **(A, B)** wild type; **(C, D)***amiR-CESA4*; **(E, F)***amiR-CESA7*. Left hand panels are observed through a 4x lens and a gray scale value of 255 indicates a retardance value of 5 nm; Right hand panels are observed through a 20x lens and a gray scale value of 255 indicates a retardance value of 13 nm. Bars = 50 μm **(E)** and 100 μm **(F)**.

To examine cellulose crystallinity on a cellular scale, we used polarized-light microscopy on plants grown at one time and used also for the sum frequency generation spectroscopy. As described in Methods, our microscope is based on circularly polarized light, allowing contrast to be independent of crystal orientation within the plane perpendicular to the microscope’s optical axis, and generating images in which intensity is proportional to birefringent retardance (Figure 9). As expected for the ubiquitous presence of cellulose, all cell walls of the wild-type had retardance, with epidermis, metaxylem, and cells of the vascular sheath being particularly strong (Figure 9A, B). In contrast, cell walls in the stem of *amiR-CESA4* had weak retardance (Figure 9C, D). Although retardance would tend to decrease along with cell wall thickness, the decrease in retardance was much larger than that of cell wall thickness. Likewise, compared to the wild-type, cell walls of *amiR-CESA7* had less retardance (Figure 9E, F). However, for this genotype the decrease was modest and within the range that might be attributable to the thinner cell walls. These results, taken together with those from diffraction and spectroscopy, confirm that the reduced expression of *BdCESA4* reduced the amount of crystalline cellulose present in the secondary cell wall.

## Discussion

For *B. distachyon,* this is apparently the first study detailing the *CESA* gene family and functionally characterizing *BdCESA*s involved in secondary cell wall synthesis. Members of the CESA family are best characterized in *A. thaliana,* where they are assigned a role for cellulose synthesis in either primary or secondary cell wall. Other vascular plants have a similar gene family structure [13]. The *B. distachyon CESA* gene family includes the uncharacterized grass-specific CESA clade (*BdCESA10*), which has been identified in all grass genomes sequenced to date, and all the other previously described clades [13,24,28].

Using amino acid sequences, the ten BdCESA genes were categorized into two groups: primary and secondary. Each of the three secondary CESA clades contains a single *A. thaliana* protein and a single rice protein, which have all been functionally characterized [17,18,23]. Fittingly, a single BdCESA protein was present in each of these clades, suggesting they have not expanded since the time of eudicot and monocot divergence 140–150 million years ago [29]. This differs for the primary CESAs, which have differentially expanded between eudicots and monocots. There are different numbers of proteins in the CESA1 and CESA3 clades in *A. thaliana*, *B. distachyon,* and rice, and the CESA6 clade has diverged into separate eudicot and monocot clades, referred to as CESA6A and CESA6B, respectively [13]. Among the three secondary *B. distachyon* CESAs, *BdCESA8* was the most divergent, as was that clade member (*OsCESA4*) in rice [23]. Moreover, *BdCESA8* and *OsCESA4* both lack eight amino acids in the first RING type zinc finger motif. However, *A. thaliana CESA8* neither lacks those eight amino acids nor is notably divergent.

Confirming the deduction from phylogeny, expression of the secondary *BdCESAs* was generally enriched in stems, which are abundant in secondary cell walls, and transcripts were specifically abundant in stem vascular tissue and the surrounding mechanical tissue including sub-epidermal cell layers, tissues that all make secondary cell walls. This is consistent with the tissue-specific patterns of secondary CESA expression observed in rice, maize, *A. thaliana,* and barley [19,24,30,31]. Interestingly, in developing stems of barley, *HvCESA8* expression was two-fold greater than that of *HvCESA4*[31], which was also observed here for *BdCESA8* and *BdCESA4*. Taken together, we conclude that *BdCESA4*, *7,* and *8* encode cellulose synthase catalytic subunits for the secondary cell wall.

To our knowledge, this is the first report of artificial microRNA-mediated gene silencing in *B. distachyon*. We characterized the loss-of-function lines generated for *BdCESA4* or *BdCESA7* for changes in morphology and cellulose crystallinity. While these lines had a small but significant delay in inflorescence emergence and a reduction in stature, whole plant morphology was similar to wild-type plants. Similarly, delayed flowering and reduced stature was observed in the *A. thaliana irx3* mutant and in rice *brittle culm* mutants [25,32]. Sections of the stem revealed that the knock-down lines had a small but significant reduction in stem internode transverse cross-sectional area, a reduction that resembles that observed in rice *brittle culm* mutants and barley *fragile stem 2*[23,33].

The loss-of-function lines for *BdCESA4* and *7* had cell walls in xylem and sclerenchyma that were modestly but significantly thinner than wild-type. Thinner cell walls are expected from a defect in secondary cell wall cellulose synthesis, and have been observed consistently in secondary cell wall *cesa* mutants [22,23,25]. In *A. thaliana*, the defining phenotype of secondary *CESA* mutants is irregular or collapsed xylem [17,18]. However, xylem contours appeared to be regular here, as they do in the secondary *CESA* mutants of rice [23]. These differences probably reflect distinctions between eudicots and monocots in xylem tension or cell wall composition rather than differences in protein function [34,35].

Cellulose is a linear glucan polymer made of β (1–4) linked glucose molecules. Thirty-six such glucan chains are predicted to form a cellulose microfibril [36]. Hydrogen bonding between microfibrils crystallizes multiple cellulose chains together providing the physical properties necessary to maintain rigid plant cell walls. Crystallinity also allows cellulose to be readily quantified. We took advantage of spectroscopic techniques (X-ray diffraction and sum frequency generation) and polarized-light microscopy to assess the status of cellulose in the loss-of-function lines.

For the *amiR-CESA7* mutants there was no significant decrease in crystallinity seen spectroscopically and the small decrease in birefringent retardance might reflect the thinner cell walls. Given that *BdCESA7* expression was reduced by less than a factor of two, a generally wild-type cellulose status is perhaps not unexpected. In contrast, the *amiR-CESA4* mutants had substantially decreased retardance and significantly decreased crystallinity from both diffraction and spectroscopic methods. The most parsimonious explanation for these data is that the cell walls of this line simply contained less cellulose than those of the wild-type. A nuanced explanation is that the cellulose synthesized in this line contained more defects, such as amorphous regions, or comprised microfibrils with fewer than the usual number (e.g., 36) of glucose chains. These alternatives are difficult to distinguish. Be that as it may, the significant decrease in crystalline cellulose amount or quality in this line matches the nearly 10-fold reduction in *BdCESA4* expression. This result implies that the function of *BdCESA4* is at least partially non-redundant and it is noteworthy that the expression of neither *BdCESA7* nor *8* was elevated in an attempt at compensation.

## Conclusion

Interestingly, even though the two lines differed in the strength of the knock-down and in the consequent loss of cellulose, the morphological defects in the plants were similar and modest. This implies that small decreases in CESA activity are sufficient to impact the plant, possibly through a feedback system analogous to the one *A. thaliana* invoking THESEUS [37], but that progressively larger changes to the plant need not follow from larger decreases in CESA activity. In this connection, it will be interesting to observe the phenotype of a complete loss of function mutant for BdCESA4.

## Methods

### Plant material and growth

*Brachypodium distachyon* (L.) line Bd 21–3 was used throughout. Seeds were imbibed in moist paper towels for seven days at 6°C, planted on potting mix (#2; Conrad Fafard Inc. Agawam, MA), and grown in a growth chamber at 20°C with 20 h light: 4 h dark, at a fluence rate of 220 μmol^.^m^-2.^s^-1^ and relative humidity of ~68%. For plate-grown plants, seeds were de-hulled and imbibed in water for 2 h with shaking. Then, seeds were treated with 70% ethanol for 20 s, rinsed with sterile water, and soaked in 1.3% NaClO for 4 min at room temperature while shaking. Seeds were subsequently rinsed three times with sterile water and stored in the dark at 4°C for a minimum of 2 days in a sterile Petri dish with filter paper. Seedlings were grown for seven days on 0.5 strength MS medium adjusted to a pH of 5.8 with KOH and containing 0.7% agar (Difco “Bacto agar”).

### Identification of *CELLULOSE SYNTHASE A* genes

Complete, translated amino acid sequences of the ten *A. thaliana* CESA proteins and members of the cellulose synthase-like families were used as queries in BLASTP of Phytozome v8.0 and NCBI databases to identify homologous *B. distachyon* CESA genes and identified proteins were named according to the *A. thaliana* counterparts, where possible. Multiple sequence alignments were performed using ClustalW program and a phylogenetic trees were generated by the neighbor-joining method using MEGA5 software with 1000 bootstrap permutations [38].

### Measurements of transcript abundance

For microarray analysis, different growth regimes were used for leaves and stems, versus roots. For leaves and stems, approximately 30 days following germination and growth on soil, total leaf and stem were collected when the inflorescence began to emerge from the flag leaf. Leaves were separated from the stems with a curved-tip probe. Nodes and internodes from the second leaf junction to the internode below the inflorescence were frozen in liquid nitrogen. For roots, seven-day-old whole seedlings were flash frozen in liquid nitrogen and then the roots were snapped off into a sterile culture tube. For all organs, material was harvested six times during the day (2, 6, 10, 14, 18, and 22 h circadian time). Three plants were dissected for each time point and in triplicate for each tissue type. Samples were stored in liquid nitrogen or at −80°C until RNA extraction. Tissue was ground with mortar and pestle in liquid nitrogen. RNA was extracted using a kit (Plant RNaeasy, Qiagen, Valencia, CA) according to the manufacturer’s instructions. For hybridization, cDNA probes were synthesized using a kit (WT Ambion Santa Clara, CA).

The probes were applied to the *B. distachyon* BradiAR1b520742 whole genome tiling array (Affymetrix, Santa Clara, CA). The array contains ~6.5 million unique 25-mer oligonucleotide features, both the forward and reverse strand sequence. The complete genome sequence is tiled with an average of 30 bases between each array feature; 1.6 million features correspond to exons and introns and 4.9 million features between gene models (Todd Mockler, Donald Danforth Plant Science Center, personal communication). Approximately ~95% (~26,670) of the genes have at least five corresponding exon array features and from those a summary value was calculated for each gene model. Probeset values were calculated using gcRMA [39].

For obtaining RNA from the transgenics, stems were collected at the same developmental stage when the inflorescence was just visible from the flag leaf. First, second, and third nodes and internodes of the tallest stem were frozen in liquid nitrogen, homogenized, and RNA extracted as described above.

For RT-QPCR, on-column DNA digestion was performed using RNase-free DNase I (Qiagen). First strand cDNA was synthesized from 1 μg of total RNA using Superscript III reverse transcriptase with oligo dT primers (Invitrogen, Grand Island, NY). Samples were diluted three-fold with RNase free water (Qiagen) and 1 μL from each cDNA sample was used for RT-QPCR to check for genomic DNA contamination using GapDH primers. Triplicate quantitative PCR reactions were performed using 20 μL reaction volumes with 1 μL of diluted cDNA in each reaction with the QuantiFast SYBR Green PCR Kit (Qiagen). The reactions were conducted in an Eppendorff Realplex^2^ Mastercycler using the following conditions: 95°C for 2 min, followed by 40 cycles of 95°C for 15 s, 60°C for 15 s and 68°C for 20 s. As reference genes for normalization, *BdUBC18* (ubiquitin-conjugating enzyme 18) and *Bd5g25870* (Belonging to nuclear hormone receptor binding category) were used [40]. QuantiPrime primer design tool was used for qPCR primer design [41].

### RNA in situ hybridization

RNA *in situ* hybridization was performed using methods described previously [42,43]. Briefly, the 3′ end of *BdCESA4* and *BdCESA7* were cloned into the pGEM-T Easy vector and used as the template to generate labeled sense and anti-sense ribo probes using a kit (DIG labeling kit, Roche, Indianapolis, IN). Three week old stem sections were frozen at −80°C and fixed in 4% paraformaldehyde/ethanol:acetic acid (3:1) overnight at 4°C [44]. Fixed tissue was encased in 4% agarose and sectioned using a Vibratome. Sections were removed from agarose using forceps and washed in phosphate-buffered saline (PBS; 33 mM Na_2_HPO_4_, 1.8 mM NaH_2_PO_4_ and 140 mM NaCl, pH 7.2) and post-fixed in PBS containing 3.7% (v/v) formaldehyde and 2 mg/mL glycine for 20 min at room temperature. Sections were washed in PBS and dehydrated in graded ethanol series and pre-hybridized for 1 h at 65°C with hybridization solution comprising 20X SSC (3M NaCl, 0.3M Na citrate) containing 20% SDS, 3.7% formamide, and 10 mg/mL yeast tRNA made up in DEPC-treated water. Stems were then hybridized with DIG-labeled sense and anti-sense probes overnight at 65°C and washed with a series of SSC and SDS containing solutions. Anti-DIG alkaline phosphatase fab fragments (Roche) were used at a dilution of 1:1000 and incubated over night at 4°C. Alkaline phosphatase was detected using nitroblue tetrazolium and 5-bromo-4-chloro-3-indoyl-phosphate (Roche) in 0.1 M Tris (pH 9.5), 0.1 M NaCl and imaged with a PixeLINK camera attached to a Nikon eclipse 200 microscope.

### Artificial microRNA constructs

Artificial microRNA sequences were designed on the Web MicroRNA Designer platform (http://wmd3.weigelworld.org) based on JGI *B. distachyon* genome annotation version 1.0 [5]. The *amiR-CESA4* construct targets AAGGGACCCATTCTTAAGCCA with hybridization energy of −37.74 kcal/mol and the *amiR-CESA7* construct targets ACGCCCACCATTGTCATCATC with hybridization energy of −37.37 kcal/mol. Constructs were engineered from the pNW55 plasmid to replace the targeting regions of the native rice microRNA precursor *osaMIR528*[45]. MicroRNA targets were PCR amplified (Additional file 5: Table S2) according to Warthmann et al. [45] and cloned into pENTR/D-TOPO (Invitrogen). Sequence confirmed clones were recombined with a modified version of the destination vector pOL001 [46], pOL001-ubigate-ori1 and transformed into *Agrobaterium tumefaciens* strain *AGL1* via electroporation.

### Plant transformation

Transformation was carried out according to Vogel et al. [47]. Briefly, seeds were collected from six to seven week old plants and deglumed. Seeds were surface sterilized with a 1.3% NaClO solution containing 0.01% Triton-X100 for 4 min. Embryos were dissected and placed on callus initiation medium under aseptic conditions. Calli were propagated for seven weeks with two subsequent subcultures at four and six weeks following dissection. Seven-week-old calli were immersed in an *A. tumefaciens* suspension for 5 min and dried on filter paper. Next, they were co-cultivated on dry filter paper for three days at 22°C in the dark. Following co-cultivation, calli were moved to selective plates containing 40 mg/L hygromycin and 200 mg/L timentin for four weeks in the dark at 28°C. Following selection, calli were moved to Linsmaier and Skoog media for regeneration at 28°C under constant light and next onto Murashige and Skoog media for root establishment under same conditions. Next they were transplanted to soil and grown as described above.

### Genomic DNA extraction and genotyping

Genomic DNA was extracted from leaves according to Csaikl et al. [48] with slight modification. Briefly, leaves were frozen in liquid nitrogen and ground using 3.2 mm diameter stainless steel metal balls (Biospec Products, Bartlesville, OK) in a ball mill (Mixer Mill, MM400, Retsch, Newtown, PA). Ground samples were incubated in DNA extraction buffer (100 mM NaCl, 50 mM Tris, 25 mM EDTA, 1% SDS, 10 mM 2-mercaptoethanol) for 10 min at 65°C. Next, they were mixed with 5 M potassium acetate and incubated on ice for 20 min and centrifuged for 10 min. DNA was pelleted by mixing the supernatant with isopropanol and centrifuging at maximum speed for 10 min followed by a 70% ethanol wash. Pelleted DNA was resuspended in 1X TE and the integrity of the samples was measured using a spectrophotometer (NanoDrop 1000, Themo Scientific, Waltham, MA). Genotyping was carried out by PCR for the Hgyromycin locus using the extracted genomic DNA as template with following cycler conditions; Initial denaturation at 98°C for 2 min, flowed by 30 cycles of 98°C for 30 s, 59°C for 30 s, 72°C for 55 s and a final extension at 72°C for 7 min. PCR confirmed positive transformants were used in subsequent experiments.

### Light microscopy

For histochemical analysis, stems were hand sectioned using a razor blade and stained with 0.002% Toluidine blue in water for 30 s. Stained sections were mounted with water and observed under an Eclipse E200MV R microscope (Nikon) and imaged using a PixeLINK 3 MP camera. Images were captured at 4 X magnification and stem area was measured by freehand tracing of a perimeter in ImageJ (http://rsb.info.nih.gov/ij/). Images captured at 20 X magnification were used for cell wall thickness measurements.

For polarized-light microscopy, internode segments were fixed in 2% glutaraldehyde in 50 mM Na_2_PO_4_ buffer (pH 7.2) for a minimum of 2 h at room temperature and then handled and embedded in Spurr’s resin by standard techniques. Semi-thin sections (0.5 μm) were cut on an ultra-microtome, mounted in immersion oil, and observed through a microscope equipped with the LC-PolScope (CRI Cambridge MA) as described previously [49]. Briefly, this instrument uses circularly polarized light and computer-controlled liquid crystal compensators to obtain four images with known compensator settings and from them calculates a fifth image in which the intensity of each pixel is proportional to birefringent retardation and a sixth image (not used here) in which the intensity of each pixel represents the orientation of the crystal’s optical axis within the specimen plane [50]. Several sections of each genotype were observed and the images shown are representative.

### X-ray diffraction profiles and sum-frequency-generation vibration spectroscopy

X-ray diffraction and calculation of a crystallinity index were done as described by Ruland et al. [51] with slight modifications. Fully senesced stems were ground as described above for genomic DNA extraction. Diffraction was analyzed on an X’Pert Pro powder X-ray diffractometer (PANanalytical BV, The Netherlands) operated at 45 kV and 45 mA using CuKα radiation at both Kα1 (λ = 1.5406 Å) and Kα2 (λ = 1.5444 Å). The diffraction profile was acquired from 5 to 50˚ in 0.0167 steps, with 66 s per step. The crystallinity index was calculated using the amorphous subtraction method, which determines crystallinity by subtracting the amorphous contribution from the diffraction profile obtained from xylan (Aldrich) measured in parallel. A scale factor was applied to the xylan profile to avoid negative values in the subtracted profiles. For each group, eight to twelve individuals were analyzed. Sum-frequency vibration spectroscopy was done as previously described [26]. Intact first internodes were excised from completely senesced plants, and each group contained four to six individuals with ten measurements per stem. The amount of crystalline cellulose was estimated by comparing the 2944 cm^-1^ intensity of the sample with that of Avicel, as previously described [27].

### Statistical analysis

For each measurement, three to twelve independent plants were sampled from three or four different T_3_ families for each transgene. Student’s *t*-tests were performed in R (v 2.15.0). Significance was set a *P* < 0.05. No significant differences were observed among the different independent transgenic lines for a given construct and were thus pooled.

### Availability of supporting data

The microarray raw data (CEL files) have been deposited at PLEXdb [Accession no: BD3] [52].

## Competing interests

The authors declare that they have no competing interests.

## Authors’ contributions

PP, KO, SH, designed the study. PP, DM, KO, MH, KH, KK, SK, TB performed experiments. PP, KH, KK, SK, SH analyzed the data. PP, TB, SH wrote the manuscript. All authors read and approved the final manuscript.

## Supplementary Material

Additional file 1: Figure S1 Phylogenetic analysis of *A. thaliana*, *B. distachyon* and rice CESA superfamily amino acid sequences. A consensus phylogeny was constructed with the neighbor-joining method with 1000 bootstrap permutations. The CESA clade is illustrated as an expanded sub-tree and the CSL clades are illustrated as condensed sub-trees. Click here for file

Additional file 2: Figure S2 Alignment of the *B. distachyon* three secondary cell wall CESA amino acid sequences. The overhead blue line shows the RING-type zinc finger motif, with cystine residues highlighted in blue. The overhead green lines show the two hypervariable regions. Pink highlights the three putatively catalytic aspartate residues and purple highlights the QxxRW motif. The overhead yellow line shows the plant-specific region and overhead black lines show the eight putative transmembrane domains. Click here for file

Additional file 3: Table S1 The ratios of relative transcripts abundance of the *CESA*s in root (R), leaf (L) and stem (S) tissue. Click here for file

Additional file 4: Figure S3 Relative expression of selected non-targeted *BdCESA* genes. Transcript abundance measured by RT-QPCR. The boxes comprise data from three to five individuals from three to four independent transgenic lines. Stem tissue was collected at the same development stage when inflorescence was just emerging from the flag leaf. Box plots and significance are as described for Figure 5. Click here for file

Additional file 5: Table S2 List of oligonucleotides used in this study. Click here for file
