# Supplementary material for: Perturbation of Brachypodium distachyon CELLULOSE SYNTHASE A4 or 7 results in abnormal cell walls
Source: BMC Plant Biol. 2013 Sep 11;13:131. doi: 10.1186/1471-2229-13-131 (PMC3847494; doi:10.1186/1471-2229-13-131)
Supplement: Additional file 1: Figure S1 — Phylogenetic analysis of A. thaliana, B. distachyon and rice CESA superfamily amino acid sequences. A consensus phylogeny was constructed with the neighbor-joining method with 1000 bootstrap permutations. The CESA clade is illustrated as an expanded sub-tree and the CSL clades are illustrated as condensed sub-trees. [file 1471-2229-13-131-S1.pptx]

## Slide 1
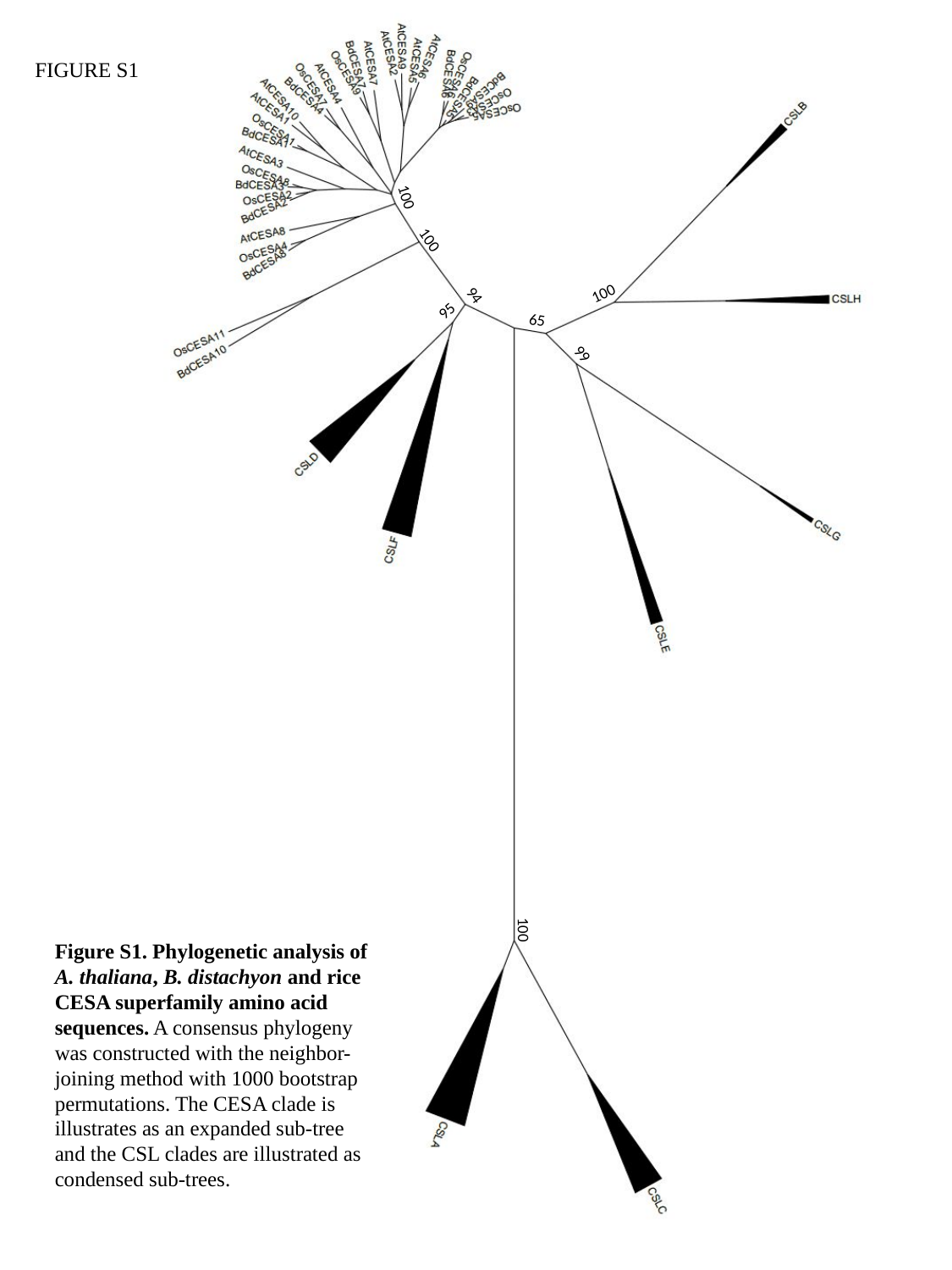

FIGURE S1
100
100
100
94
95
65
99
100
Figure S1. Phylogenetic analysis of A. thaliana, B. distachyon and rice CESA superfamily amino acid sequences. A consensus phylogeny was constructed with the neighbor-joining method with 1000 bootstrap permutations. The CESA clade is illustrates as an expanded sub-tree and the CSL clades are illustrated as condensed sub-trees.
