# Supplementary material for: Perturbation of Brachypodium distachyon CELLULOSE SYNTHASE A4 or 7 results in abnormal cell walls
Source: BMC Plant Biol. 2013 Sep 11;13:131. doi: 10.1186/1471-2229-13-131 (PMC3847494; doi:10.1186/1471-2229-13-131)
Supplement: Additional file 2: Figure S2 — Alignment of the B. distachyon three secondary cell wall CESA amino acid sequences. The overhead blue line shows the RING-type zinc finger motif, with cystine residues highlighted in blue. The overhead green lines show the two hypervariable regions. Pink highlights the three putatively catalytic aspartate residues and purple highlights the QxxRW motif. The overhead yellow line shows the plant-specific region and overhead black lines show the eight putative transmembrane domains. [file 1471-2229-13-131-S2.pptx]

## Slide 1
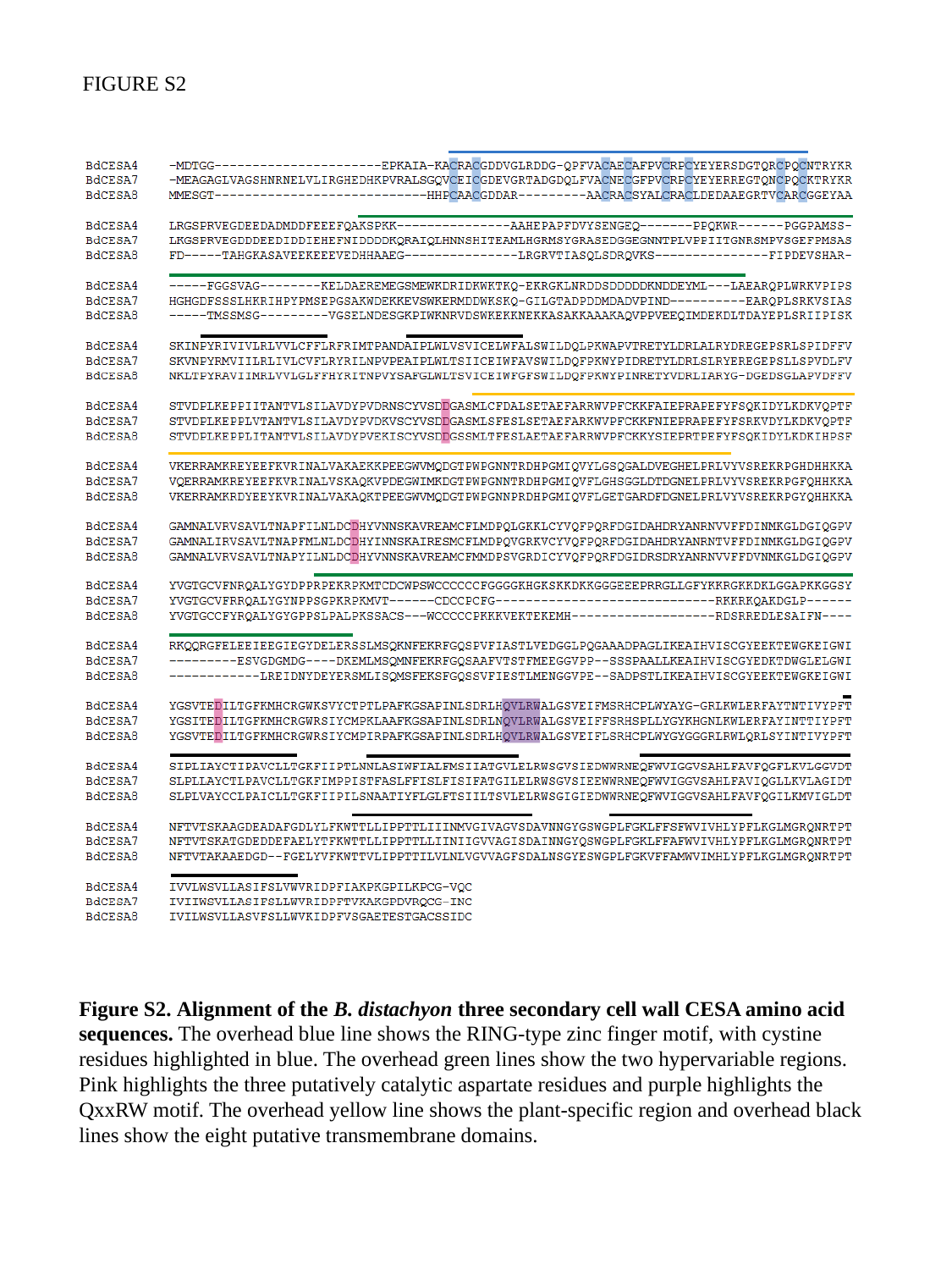

FIGURE S2
Figure S2. Alignment of the B. distachyon three secondary cell wall CESA amino acid sequences. The overhead blue line shows the RING-type zinc finger motif, with cystine residues highlighted in blue. The overhead green lines show the two hypervariable regions. Pink highlights the three putatively catalytic aspartate residues and purple highlights the QxxRW motif. The overhead yellow line shows the plant-specific region and overhead black lines show the eight putative transmembrane domains.
