# Supplementary material for: Perturbation of Brachypodium distachyon CELLULOSE SYNTHASE A4 or 7 results in abnormal cell walls
Source: BMC Plant Biol. 2013 Sep 11;13:131. doi: 10.1186/1471-2229-13-131 (PMC3847494; doi:10.1186/1471-2229-13-131)
Supplement: Additional file 3: Table S1 — The ratios of relative transcripts abundance of the CESAs in root (R), leaf (L) and stem (S) tissue. [file 1471-2229-13-131-S3.pptx]

## Slide 1
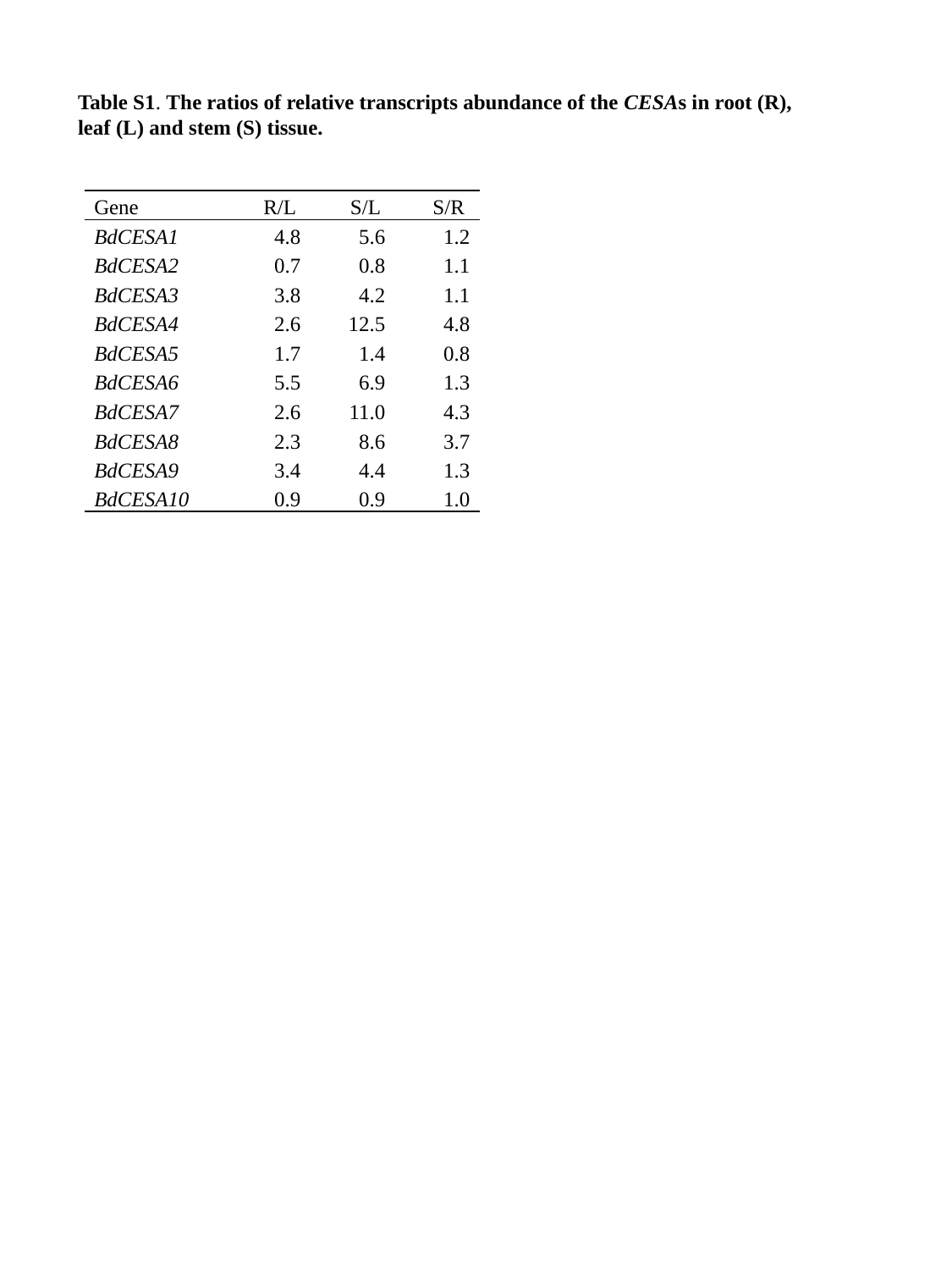

Table S1. The ratios of relative transcripts abundance of the CESAs in root (R), leaf (L) and stem (S) tissue.
| Gene | R/L | S/L | S/R |
| --- | --- | --- | --- |
| BdCESA1 | 4.8 | 5.6 | 1.2 |
| BdCESA2 | 0.7 | 0.8 | 1.1 |
| BdCESA3 | 3.8 | 4.2 | 1.1 |
| BdCESA4 | 2.6 | 12.5 | 4.8 |
| BdCESA5 | 1.7 | 1.4 | 0.8 |
| BdCESA6 | 5.5 | 6.9 | 1.3 |
| BdCESA7 | 2.6 | 11.0 | 4.3 |
| BdCESA8 | 2.3 | 8.6 | 3.7 |
| BdCESA9 | 3.4 | 4.4 | 1.3 |
| BdCESA10 | 0.9 | 0.9 | 1.0 |
