# Supplementary material for: Perturbation of Brachypodium distachyon CELLULOSE SYNTHASE A4 or 7 results in abnormal cell walls
Source: BMC Plant Biol. 2013 Sep 11;13:131. doi: 10.1186/1471-2229-13-131 (PMC3847494; doi:10.1186/1471-2229-13-131)
Supplement: Additional file 4: Figure S3 — Relative expression of selected non-targeted BdCESA genes. Transcript abundance measured by RT-QPCR. The boxes comprise data from three to five individuals from three to four independent transgenic lines. Stem tissue was collected at the same development stage when inflorescence was just emerging from the flag leaf. Box plots and significance are as described for Figure 5. [file 1471-2229-13-131-S4.pptx]

## Slide 1
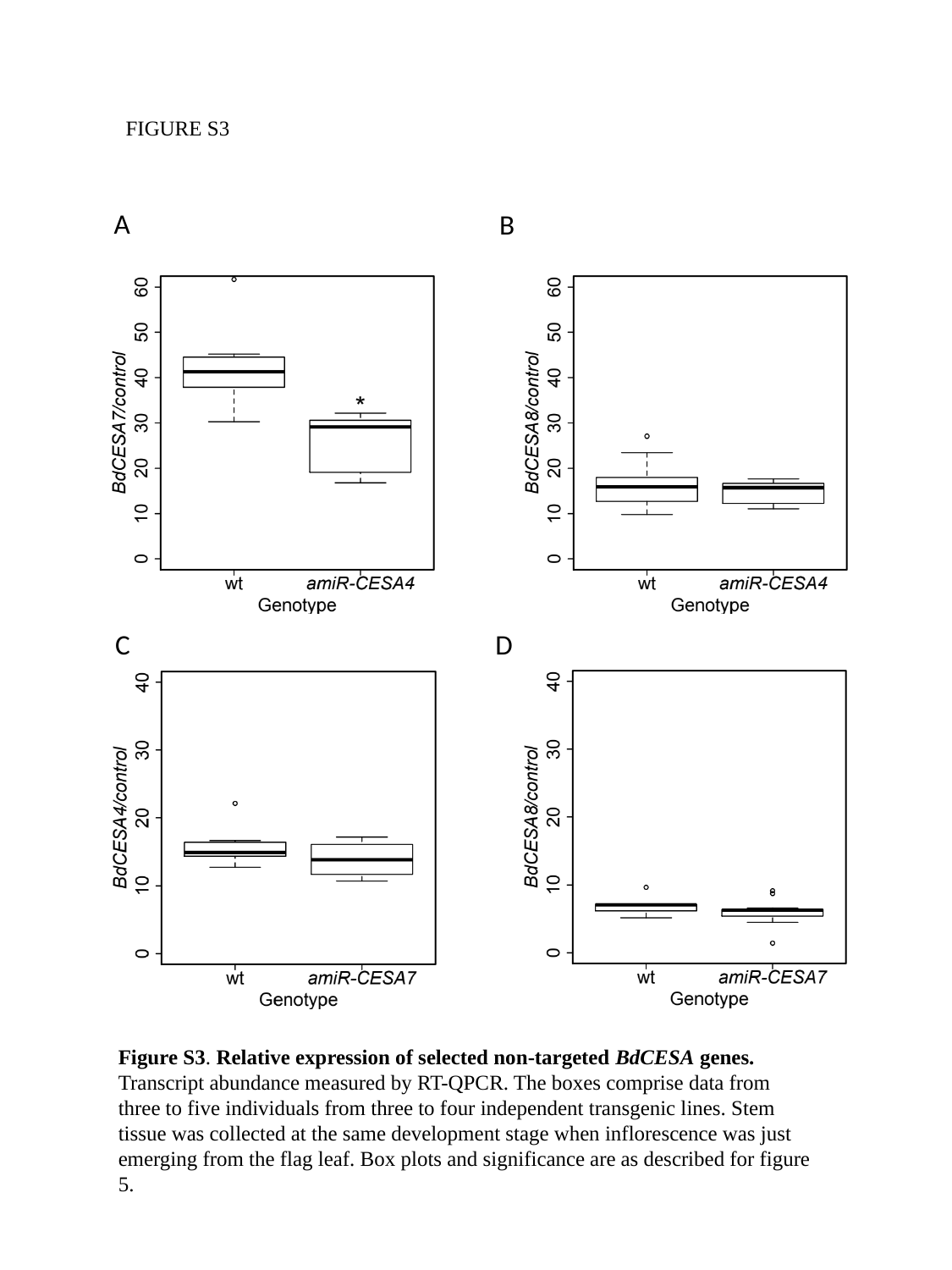

FIGURE S3
A
B
C
D
Figure S3. Relative expression of selected non-targeted BdCESA genes. Transcript abundance measured by RT-QPCR. The boxes comprise data from three to five individuals from three to four independent transgenic lines. Stem tissue was collected at the same development stage when inflorescence was just emerging from the flag leaf. Box plots and significance are as described for figure 5.
