# Supplementary figures and images for: Perturbation of Brachypodium distachyon CELLULOSE SYNTHASE A4 or 7 results in abnormal cell walls
Source: BMC Plant Biol. 2013 Sep 11;13:131. doi: 10.1186/1471-2229-13-131 (PMC3847494; doi:10.1186/1471-2229-13-131)

## Slide 1
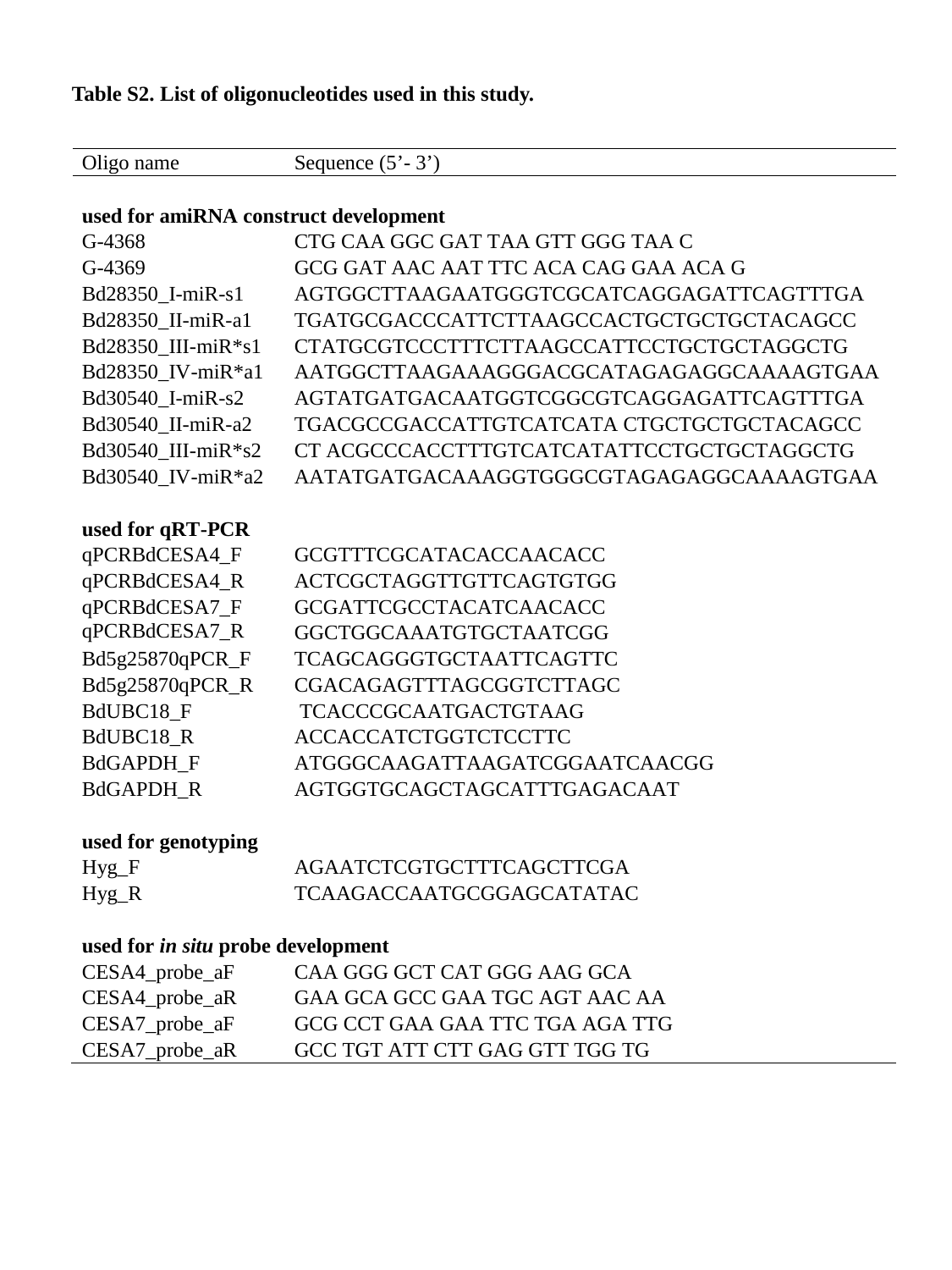

Table S2. List of oligonucleotides used in this study.

Supplement: Additional file 5: Table S2 — List of oligonucleotides used in this study. [file 1471-2229-13-131-S5.pptx]
